# An investigation of economic costs of schizophrenia in two areas of China

**DOI:** 10.1186/1752-4458-7-26

**Published:** 2013-11-15

**Authors:** Jinguo Zhai, Xiaofeng Guo, Min Chen, Jingping Zhao, Zhonghua Su

**Affiliations:** 1 School of Mental Health, Jining Medical University, No. 16, Lotus Road, Jining 272067, Shandong Province, PR China; 2 Institute of Mental Health of the Second Xiangya Hospital, Central South University, No.139 Renmin Middle Road, Changsha 410011, Hunan, PR China; 3 Department of Psychiatry, the Second Affiliated Hospital, Jining Medical University, Jining 272051, Shandong, PR China

**Keywords:** Schizophrenia, Economic burden, Economic cost

## Abstract

**Background:**

Schizophrenia is a severe psychotic disorder characterized by significant disturbances in thinking, perception, emotions and behavior. Even if it is not a very frequent disorder, but it is the most burdensome and costly illnesses worldwide. The total population was approximate 1.3 billion and there are approximate 8 million schizophrenic patients in China. Despite the wide-ranging financial and social burdens associated with schizophrenia, but there have been few cost-of-illness studies of this illness in China.

**Objective:**

To evaluate the economic cost of schizophrenic patients in China.

**Methodology:**

356 schizophrenic patients who met with DSM-IV criteria were enrolled and investigated with the Economic Burden Questionnaire(EBQ), 299 schizophrenic patients completed the study for 12 months. All the data were combined and classified by researcher. EBQ include all kinds of cost such as direct cost, indirect cost and total cost as well. It was filled in by patients and their close caregivers. Comparison of cost was made between not only out-patients and in-patients but also urban patients and rural patients. Multiple stepwise regression analysis was made to identify the main influence factors of total cost.

**Results:**

(i) The per case per annum total costs, direct costs and indirect costs of schizophrenia amounted to US$2586.21, US$862.81(33.4%) and US$1723.40(66.6%) respectively. The per case total cost, direct cost and direct medical cost of in-patients were more higher than out-patients (*P* < 0.05). (ii) There was significant difference in per case per annum total cost, direct cost, direct medical cost, cost due to lost working-days and disability between urban and rural schizophrenic patients (*P* < 0.05), the former is higher than the latter. (iii) The results of multivariate stepwise regression analysis show that five variables were significantly correlated with higher cost: professional status(cadre), diagnostic subtype(residual schizophrenia), urban or rural patients(urban patients), in-patients or out-patients(in-patients) and researcher centre(southern center). The standardized regression coefficient were 0.308, 0.218, 0.212, 0.156 and 0.149 respectively, the correlation of determination R square was 0.2741, F = 15.651, P < 0.0000. These characteristics explain 27.41% of the variability in the total cost.

**Conclusion:**

(i) Economic cost of schizophrenia were serious, we must pay close attention to it. (ii) The indirect cost are the majority of the total cost. The cost of urban patients are more higher than the cost of rural patients, the cost of in-patients are more higher than the cost of out-patients.

## Introduction

Schizophrenia is a chronic, devastating mental disorder characterized by distortions of thinking and perception. About 1.1 percent of the population age 18 and older have schizophrenia. It is very costly for families and society. The expensive costs of schizophrenia imposes a disease burden on themselves, health care providers, their families and the wider society [1-4]. The total economic burden of schizophrenia is comparable to the total costs of any other mental disorder. In most of the developed countries, current healthcare expenditure on schizophrenia accounts for 1.6% to 2.5% of the total healthcare budget [5,6]. Schizophrenia is a chronic mental illness associated with a significant and long-lasting health, social, and financial burden due to expenditures for hospitalization, treatment and rehabilitation, reduced and lost productivity [5-10].

The treatment for schizophrenia is still a major clinical challenge to psychiatrists. The characterized psychotic symptoms of schizophrenia such as hallucination and delusion, inappropriate affect, loss of association induced the treatments more complex. The prognosis of schizophrenia is poor for at least one third of patients. Schizophrenia patients have a higher risk of comorbidity and social exclusion and the mortality rate is more than twice that in the overall population. As a result, schizophrenia is the most costly psychiatric illness currently [10]. An Italian study showed that the monthly service costs per patient was nearly double that for patients with other diagnoses. Non-service costs associated with patients’ lack of job opportunities were more than three times higher for schizophrenic patients [11].

International cost of illness (COI) studies for schizophrenia show a wide variation of annual costs [12-16]. Mangalore’s study investigated the costs of schizophrenia in England in 2004/2005. The estimated total societal cost of schizophrenia was 6.7 billion pounds. The direct cost of treatment and care was about 2 billion pounds; the indirect cost to the society was huge, amounting to nearly 4.7 billion pounds. The cost of lost productivity due to unemployment, absence from work and premature mortality of patients was 3.4 billion pounds. Cost of informal care and private expenditures borne by families was 615 million pounds. The cost of lost productivity of caregivers was 32 million pounds. Estimated cost to the criminal justice system was about 1 million pounds. It is estimated that about 570 million pounds will be paid out in benefit payments and the cost of administration associated with this is about 14 million pounds [12].

In Canada, Goeree used a prevalence-based cost-of-illness (COI) approach to estimate the financial burden of schizophrenia in 2004. Results showed that the direct healthcare and non-healthcare costs were estimated to be 2.02 billion Canadian dollars (CAN dollars) in 2004. There were 374 deaths attributed to schizophrenia. The cost of lost productivity due to unemployment, absence from work and an additional productivity morbidity estimate of 4.83 billion CAN dollars, for a total cost estimate of 6.85 billion CAN dollars. The largest part of the total cost estimate was for productivity losses associated with morbidity in schizophrenia (70% of total costs). Their results also showed that total cost estimates were most sensitive to alternative assumptions regarding the additional unemployment due to schizophrenia [13].

In Australia [14], Carr estimated the costs of psychosis, most of the patients were schizophrenic patients. His results showed that annual societal costs for the average patient are of the order of 46,200 Australian dollars, 27,500 Australian dollars in lost productivity, 13,800 Australian dollars in inpatient mental health care costs and 4900 Australian dollars in other costs. The total economic cost in Australia was at least 1.45 billion Australian dollars per annum. The societal costs are at least 2.25 billion Australian dollars per annum. The societal cost due to schizophrenia was at least 1.44 billion Australian dollars.

Schizophrenia accounts for 2.5% of total health care expenditures in the US. The cost of all mental illness in the US has been estimated at US$103.7 billion, of which schizophrenia alone accounts for US$22.7 billion [15].

Lee investigated the economic costs of outpatients with schizophrenia in Taiwan China, and surveyed the influence factors of costs. The average annual total cost was approximately US$16,576 per patient. The direct and indirect costs were 13% and 87% of the total costs, respectively, the indirect costs were higher than the direct costs. The productivity loss of both the patients and their caregivers was the major component of the indirect costs. The main influence factors of costs were patient’s age, global functions, severity of extrapyramidal symptoms, type of antipsychotics and patient’s illness duration [16].

The total population was approximate 1.3 billion in China. There are approximate 8 million schizophrenic patients in China [17]. Despite the wide-ranging financial and social burdens associated with schizophrenia, there have been few cost-of-illness studies of this illness in China.

The present paper aims at: (i) evaluating per case per annum total economic costs, direct costs and indirect costs of schizophrenia using a bottom-up approach; (ii) comparing the differences of the cost between in-patients and out-patients; (iii) comparing the difference of the cost between urban-patients and rural-patients; (iv) empirically determining the explanatory variables of total cost variations. The final purpose being that of offering useful information to policies makers and highlighting the economic importance of schizophrenia.

## Materials and methods

### Study design and patients

The present study is a prospective prevalence-based [12,18] multi-centre COI observational study analyzing costs in a sample of patients with schizophrenia in two areas of China (main land). The period of study was of 12 months and referred to 2010. Two centers took part in the project; one from northern China and another from southern China. All participants were out-patients or in-patients with schizophrenia from the two centers. The northern researcher centre is an affiliated hospital of Jining Medical University in Shandong Province, it’s main catchment area is Shandong Province and surrounding area. The southern researcher centre is an affiliated hospital of Central South University in Hunan Province. It’s main catchment area is Hunan Province and surrounding area. The two centers were all psychiatric hospitals treating various mental disorders and covered by similar insurance model of China.

Patients eligible for the study had a diagnosis of schizophrenia, defined according to the Diagnostic and Statistical Manual of Mental Disorders-fourth edition (DSM-IV) criteria.

Each centre required to recruit patients, meeting the inclusion and exclusion criteria.

Inclusion criteria were:

i. Meet with the diagnostic criteria of schizophrenia of DSM-IV;

ii. Age between 16 and 65 years old;

iii. There is one caregiver at least and knew well the information of the illness, treatment and costs during the period of study;

iv. No plan to emigrate during the period of study;

v. Age of caregiver is more than 18 years old,educational time is more than 5 years, caregiver can understand the content of the Economic Burden Questionnaire (EBQ);

vi. Informed consent.

Exclusion criteria were:

i. With serious physical disease;

ii. Information is not particular or not credible;

iii. Plan to emigrate in 1 year;

iv. The caregiver is suffering from mental disorder;

v. Drug or alcohol abusers.

Each of the centers provided the list of all patients meeting the inclusion and exclusion criteria. Researchers selected patients randomly according to random number chart and inclusion criteria. 356 schizophrenic patients were enrolled and 299 schizophrenic patients completed the study and their data was integrated. 57 case of patients drop out for different reasons, 28 revoked the informed consent, 12 had concurrent illness, 5 emigrated, 12 could not provide complete information.

### Cost estimates

Specifically instruments EBQ were design to collect data and measure costs. EBQ consisted of Part A and Part B, Part A gathered data on: (1) socio-demographic data referred to gender, age and marriage as well; and (2) clinical data referred to duration of illness, diagnostic subtype, time in contact with hospital, previous hospitalizations in mental hospitals as well. Part B gathered data on: economic costs and time costs and other resources use as well.

Part A was filled by psychiatrists who followed up the patients during the period of study. Part B was submitted by psychiatrist to the patients’ main caregiver (usually a close relative) who filled Part B.

### Cost components

The total cost components included in the present study can be classified as direct costs and indirect costs.

Direct costs components concerned:

i. Direct medical costs--treatment costs and other costs of medical services;

ii. Direct non-medical costs--all other resource use related to a disease.

iii. Indirect costs components concerned:

 (i) Value of damaged properties;

 (ii) The costs due to lost working-days and disability incurred by patients and caregivers;

 (iii) Mortality costs--loss of productivity to suicide and related mortality.

### Measurement of costs

A bottom-up approach [12,18,19] was used to measure costs data on resources use were gathered prospectively. As to the indirect costs, the human capital approach(HCP) [18] was used to estimate production losses due to illness. The dead cases because of schizophrenia were collected and analyzed, The dead cases for other reasons were eliminated. We used the average wages of local population to evaluate losses of production.

### Analysis of data

To analyze per case per annum total costs, direct costs, indirect costs and the proportion of each part. To compare the differences of the cost not only between in-patients and out-patients but also between urban-patients and rural-patients, to determine the explanatory variables of cost variations. As variables were not normally distributed, we used the non-parameter Mann–Whitney U test.

We set up a multi-variable regression model to identify the factors that could explain the variability of average total cost of a patient. As regressions of total cost we used the following socio-demographic and medical variables recorded in our questionnaire.

Demographic variable (sex, age, episode age, marital status, etc.);

Socio-economic variable (family status, education, professional status, etc.);

Geographic variable;

Clinical variable and variable related with illness (diagnostic subtype, duration of illness, in-patient or out-patient, urban-patient or rural-patient, etc.).

Because total cost had a slightly skewed distribution, a logarithmic transformation was used in analyzing the relationship between total cost and the other independent variables. Thus the dependent variable presented an approximately normal distribution. Polytomous variables (i.e. discrete variables which have more then two options) like occupational status, educational status and diagnostic subtype were transformed into dummy variable.

All variables were entered into a stepwise backward regression model, the final model being that which offered the best explanation for total costs, using the set of available variables.

Statistical analysis was performed with SPSS 12.0 for Windows Statistical Package.

## Results

### Sample characteristics

Sample characteristics are presented in Table 1.

**Table 1:** Sample characteristics

**Variable**		**Number of cases**	**% or (**$\bar{x}$**±SD)**
Gender	Male	181	60.5
	Female	118	39.5
Education	Primary	46	15.
	Junior	131	43.8
	High school	90	30.1
	College/university	32	10.7
Marriage	Married	110	36.8
	Unmarried	150	50.2
	Others	39	13.0
Professional status	Peasant	115	38.5
	Worker	65	21.7
	Unemployed	34	11.4
	Student	32	10.7
	Intellectual	21	7.0
	Cadre	11	3.7
	Others	21	7.0
Family economic condition	Poor	54	18.1
	Middle	192	64.2
	Good	53	17.7
In /out-patient	Out- patient	143	47.8
	In-patient	156	52.2
Urban/rural patient	Urban patient	118	36.1
	Rural patient	181	63.9
Diagnostic subtype	Paranoid	217	72.6
	Disorganized	5	1.7
	Catatonic	9	3.0
	Undifferentiated	53	17.7
	Residual	15	5.0
Center	Northern	162	54.2
	Southern	137	45.8
Age	-	16 ~ 65 years	(31.41 ± 11.33) years
Episode age	-	12 ~ 61 years	(25.45 ± 9.83) years
Total duration	-	12 ~ 480 months	(71.39 ± 76.26) months
Latest duration	-	0.50 ~ 322 months	(45.45 ± 61.49)months
Episode times	-	1 ~ 13	(1.78 ± 1.70)
Family income per annum (US$)	-	147.72 ~ 35453.14	2984.96 ± 3197.70

### Total cost, direct cost and indirect cost

The per case per annum total cost, direct cost and indirect cost are shown in Table 2.

**Table 2:** The per case per annum total cost, direct cost, indirect cost (US$)

**Costs components**	$\bar{x}$**±SD**	**% of total costs**
Total cost	2586.21 ± 1343.08	100
1. Direct cost	862.81 ± 739.74	33.4
(1) Direct medical cost	714.86 ± 706.64	27.6
(2) Direct non-medical cost	147.95 ± 153.36	5.8
2. Indirect cost	1723.40 ± 1056.90	66.6
(1) Value of damaged properties	49.63 ± 109.61	1.9
(2) Cost due to lost working-days and disability	1673.77 ± 1048.42	64.7
Patients	1009.84 ± 708.21	39.1
Caregivers	663.93 ± 734.79	25.6
(3) Mortality cost	0(no suicide case)	0

Average annual total cost per patient was of US$ 2586.21, the per case per annum direct cost and indirect cost amounted to US$862.81 (33.4%) and US$1723.40 (66.6%) respectively. Direct medical cost was US$714.86, it was the great majority of direct cost, direct non-medical cost was only 5.8% of total cost. Cost due to lost working-days and disability was the great majority of indirect cost. Indirect cost can be split up patients’ (39.1%) and caregivers’ (25.6%) cost. We have not found suicide cases because of the researcher methods, objective reasons and the traditional idea of Chinese and culture background in China, hence mortality cost was 0. Value of damaged properties was US$49.63 (1.9%).

### Comparison of costs between out-patients and in-patients

The comparison of costs between out-patients and in-patients are given in Table 3.

**Table 3:** **Comparison of cost between out-patients and in-patients(US$,**$\bar{x}$**±SD)**

**Costs components**	**Out-patients (*n*** **= 156)**	**In-patients (*n*** **= 143)**	**U value**	***P *** **value**
Total cost	2008.08 ± 1012.00	3116.17 ± 1392.41	5437.50	0.000
1. Direct cost	406.38 ± 311.13	1281.21 ± 771.36	2149.50	0.000
(1) Direct medical cost	267.70 ± 255.66	1124.77 ± 739.30	1568.00	0.000
(2 ) Direct non-medical cost	138.68 ± 114.58	156.44 ± 161.36	10694.00	0.538
2. Indirect cost	1601.70 ± 956.10	1835.00 ± 1133.11	10004.00	0.124
(1) Value of damaged properties	65.49 ± 128.86	35.11 ± 86.28	10018.00	0. 108
(2) Costs due to lost working days and disability	1536.21 ± 956.77	1799.86 ± 1114.08	9694.00	0.051
Patients	936.48 ± 647.62	1077.09 ± 755.34	10079.50	0.150
Caregivers	599.73 ± 702.85	722.77 ± 760.37	10031.00	0.132

With non-parameter Mann–Whitney U test, we found that the per case total cost, direct cost and direct-medical cost of in-patients were more higher than out-patients (*P* < 0.05). There was no difference in other cost between in-patients and out-patients (*P* > 0.05).

### Comparison of cost between urban-patients and rural-patients

Comparison of cost between urban-patients and rural-patients are given in Table 4.

**Table 4:** **Comparison of cost between urban-patients and rural-patients(US$,**$\bar{x}$**±SD)**

**Costs components**	**Rural-patients (*n*** **= 181)**	**Urban-patients (*n*** **= 118)**	**U value**	***p *** **value**
Total cost	2383.90 ± 1184.40	2761.97 ± 1447.91	9333.00	0.017
1. Direct cost	776.71 ± 731.84	937.61 ± 740.70	9237.50	0.012
(1) Direct medical cost	638.61 ± 699.67	781.11 ± 708.16	9272.50	0.013
(2) Direct non-medical cost	138.10 ± 152.69	156.50 ± 153.91	10450.00	0.369
2. Indirect cost	1607.19 ± 927.49	1824.37 ± 1151.03	9941.50	0.114
(1) Value of damaged properties	40.69 ± 87.94	57.41 ± 125.20	10678.50	0.531
(2) Costs due to lost working days and disability	1566.50 ± 901.61	1766.96 ± 1157.76	10101.50	0.172
Patients	878.54 ± 584.02	1123.91 ± 784.75	9093.50	0.007
Caregivers	687.96 ± 718.58	643.05 ± 750.21	10314.50	0.279

With non-parameter Mann–Whitney U test, we found that the per case total cost, direct cost, direct medical cost and cost due to lost working day and disability of urban-patients were more higher than rural-patients (*P* < 0.05). There was no difference in other cost between in-patients and out-patients (*P* > 0.05).

### Service utilization of per patient

Direct medical cost of per out-patient visit was US$ 57.57, average annual visit times were 5, direct medical cost of in-patient per day was US$21.91, average annual hospitalization days were 34, 212 working-days were lost in one year (patient 129 days and caregivers 83 days).

### Multivariate regression analysis

Table 5 shows the results of multivariate regression analysis. Five variables were significantly correlated with higher cost: professional status (cadre), diagnostic subtype (residual schizophrenia), urban/rural patients (urban patients), in-patients/ (in-patients), and researcher centre (southern center). The standardized regression coefficient were 0.308, 0.218, 0.212, 0.156 and 0.149 respectively, the correlation of determination R square was 0.2741, F = 15.651, P < 0.0000. These characteristics explain 27.41% of the variability in the total cost.

**Table 5:** **Multivariate regression model: impact on total cost**^**a**^

**Independent variables**	**Unstandardized coefficients**	**Std. error**	**Standardized coefficients**	***t *** **value**	***P *** **value**
Professional status	0.033	0.006	0.308	5.336	0.000
Diagnostic subtype	0.037	0.009	0.218	4.256	0.000
Urban/rural patients	0.098	0.027	0.212	3.681	0.000
In-patients/out-patients	0.072	0.024	0.156	3.052	0.002
Researcher centre	0.074	0.025	0.149	2.899	0.004
Constant	3.752	0.056	-	67.213	0.000

^a^*R* square, 0.274; *F*, 15.651; *P* < 0.0000; *n* = 299.

## Discussion

The burden of schizophrenia is large and multifaceted. Economic costs are one of the most important components. This study provided an overall estimate of the costs of schizophrenia, but it can not capture all costs, as noted by other researchers, two types of the costs were always underestimated: the cost to families and the costs of publicly owned capital.

In the present study, the sample were selected from two areas, one of which was from northern China and another was from southern China, the two centers were not selected randomly, hence, the results of the study reflected only the cost of schizophrenia in two areas of China. Centers taking part in were located in the north and south of China, but none in the west and east of the country. The sample seems not to be representative of the cost of schizophrenic patients in China (mainland). The sample was not quite large, only patients, not centre, were randomly selected, and only two centers were selected. We have not found suicide cases, however, the cost due to suicide are the important parts.

We study the economic cost of schizophrenia with human capital approach(HCP), however, HCP has many limitations, it has many assumptions that are not likely to hold, e.g. it assumes that there is full employment. Goeree [20] valuated the productivity costs due to premature mortality and compared the human capital approach and friction cost (FC) methods for schizophrenia.This study showed that productivity-cost estimates from the human capital approach are substantially higher than those obtained from the FC method. In circumstances of unemployment, the human capital approach is an overestimate of future productivity losses for premature mortality. All the limitations mentioned-above must be borne in mind.

Despite these limitation, the results from the present study show that schizophrenia cause a substantial economic burden to healthcare systems, community, other caregivers and society. The per case per annum total cost, direct cost and indirect cost amounted to US$2586.21, US$862.81 (33.4%) and US$1723.40 (66.6%) respectively. Indirect costs were the great majority of the economic cost of schizophrenia. In the indirect cost, cost due to lost working days and disability were the great majority. Schizophrenia is correlated to loss of working days, lack of well-being and poor levels of social functioning. Theses are the main results drawn from the analysis of indirect costs that appear to be exclusively borne by patients, caregivers and ultimately society as a whole. These findings are consistent with the majority of results of other cost of illness studies on schizophrenia where indirect costs vary from 48% to 86.5% of total cost [21-24]. Hence we must pay close attention to the indirect of the cost of schizophrenia, take effective measure to improve the social function and reduce the time cost and other indirect cost.

China is a large developing country, the per case pure income of rural residents was about US$450, about 60% patients of our sample were peasants, students or unemployed, the total cost, amounted to US$2586.21, was heavy for them and their families who had been very poor because of the chronic illness.

A study carried out by Andlin-Sobock [19] shown that the cost per case of schizophrenia differs significantly across the different country in Europe, range from €2360 (Estonia) to €13862 (Switzerland). In every country, the cost per case of schizophrenia was higher than other mental illness. Their findings shown that the cost per case of schizophrenia differs between countries and are highest in countries with the highest national income and healthcare expenditure. This explains the estimated lower cost per case in China. In comparison with European countries, condition with relatively low cost, in schizophrenia, was associated with the lower level of economy development.

The total cost, direct cost and direct medical cost of per case of in-patients was higher than out-patients of schizophrenia. The reason, might be stated that the medical model in China was different from other countries. There are various medical model for the management of schizophrenia such as day-care center, day-hospital, patients’home, community mental health centers (CMHCs), sheltered workshops, psychiatric ambulatories and national health service as well in developed countries [18]. However it is not the same case in China, patients have to stay at home to take medicine except for hospitalization in mental hospital and seeing doctor in out-patient, have no other choice. During hospitalization in mental hospital patients could receive various kinds of treatments, hence the direct medical cost, direct cost, total cost will increase. During non-hospitalization patients receive no treatment except for drug therapy, hence all kinds of cost will decrease.

There was significant difference in per case per annum total cost, direct cost, direct medical cost, cost due to lost working-days and disability of patients between urban and rural schizophrenic patients, the former is higher than the latter. In China, expensive new antipsychotics were mainly applied to urban patients but few to rural patients, drug cost was the main part of direct medical cost to a turn, lead to the increase of direct cost and total cost. Cost due to lost working days and disability of urban patients are higher than rural patients because the economic income of urban residents was higher than rural residents in China.

Multivariate regression analysis shows that cadre professional, paranoid type schizophrenia, urban patients, in-patients and southern researcher centre were correlated with higher total costs.

The reason why the cost of urban patients and in-patients were higher is in line with the explanation above. Cadres (civil servants) are a special population with higher economic level in China. In general, their income was higher than peasants and people being unemployed, they would pay more money to the treatment of schizophrenia. It is differs from the finding of Percudani in Italy [11,25], they found being unemployed was associated with higher service cost. Percudani also found that the total cost per patients differ wildly according to whether patients had been hospital during the observation period. Patients with a previous psychiatric contact and a longer duration of illness were more costly than the other patients [11,25].

Similar with cadres (civil servants), most urban schizophrenic patients had regular income, good family economic condition, more medical knowledge and saw doctros in the early stage of the illness. They usually took new and expensive medicine. Conversely, most rural patients were peasants or people being unemployed, they had lower economic income and poor family economic condition, they could not afford to enormous economic pressure. These patients could not be hospitalized in time and they usually took cheaper medicine, so their costs were lower. Residual type schizophrenic patients had longer duration of illness and poor prognosis, with more difficulties in treatment and rehabilitation and higher probability of hospitalization, so their costs were higher.The reason why southern researcher centre correlated with higher costs was complex. The main reason was associated with economic development.

Economic cost was a complex index which was affected by many factors, not just related with the medical factors. In addition to the five factors getting into the regression equation, there were a lot of other factors influencing the economic cost of the schizophrenic patients. So the five factors can only explain the dependent variable (total economic costs) 27.41%, other factors and accidental factors accounted for 72.59%.

## Conclusion

The major strengths of this study are that of having clearly showed that (i) the economic burden of schizophrenia was serious in China, (ii) the highest proportion of the economic cost of schizophrenia was indirect cost, the cost of urban patients are more higher than the cost of rural patients, the cost of in-patients are more higher than the cost of out-patients; (iii) We must pay close attention to the economic burden of schizophrenia.

## Competing interests

The authors declare that they have no competing interests.

## Authors’ contributions

JZ, XG and MC contributed equally to the study. JZ, MC, ZS and XG carried out the collection and analysis of data, JZ and ZS contributed in drafting the manuscript. JZ contributed to the design of the study. JZ had full access to all the data in the study and takes responsibility for the integrity of the data and the accuracy of the data analysis. All authors read and approved the final manuscript.
